# Tottenham Hospital

**Published:** 1902-08-16

**Authors:** 


### TOTTENHAM HOSPITAL.

THE YEAR'S WORK.

The number of patients at this hospital is increasing, and in view of the workmen's dwellings being constructed by the London County Council at Tottenham to relieve the congested districts of London, a special appeal is being made for increased support. "What is required," says the report, "is a hospital not of 80 beds only, but of at least 400." There were 713 in-patients in 1901, the average cost per week being £1 8s. 10d. The out-patients numbered 14,789, the average cost of each being 1s. 6d. In 1900 there were 628 in- and 11,634 out-patients. The daily average number of beds occupied during 1901 was 45; the average stay of each patient was 23 days, a reduction of one day on the previous year. The ordinary income was £4,431 11s. 5d.; extraordinary, £5,643 2s. 6d. The ordinary expenditure was £4,499 12s. 8d.; extraordinary (building improvements and purchase of Consols) £6,318 11s. 11d. King Edward's Fund gave £1,000; the Sunday Fund, £248 6s. 8d.; and the Saturday Fund, £121 10s. The penny collection amounted to £136 0s. 9d.

